# Assessing the bidirectional causal relationship between malnutrition, self-reported fatigue, and stroke: A 2-sample Mendelian randomization study

**DOI:** 10.1097/MD.0000000000046894

**Published:** 2026-01-02

**Authors:** Min Luo, Shiyu Wen, Huiping Jiang, Yu Lei, Minyue Sun

**Affiliations:** a Department of Neurology, Mianyang Central Hospital, School of Medicine, University of Electronic Science and Technology of China, Mianyang, China.

**Keywords:** malnutrition, Mendelian randomization, stroke, weakness

## Abstract

This study aims to investigate the causal relationships between malnutrition, fatigue, and stroke (including its subtypes) using a 2-sample bidirectional Mendelian randomization (MR) analysis. This study utilized publicly available Genome-Wide Association Study summary statistics from the Genome-Wide Association Study Catalog and the MEGASTROKE consortium. A 2-sample MR approach was employed to explore the causal effects of malnutrition and fatigue on stroke risk. Instrumental variables were rigorously selected based on strict criteria. The primary analysis used the inverse-variance weighted method, supplemented by weighted median, MR Egger regression, and other MR methods. Sensitivity analyses, including Cochran *Q* test, MR Egger intercept test, and MR-PRESSO, were conducted to assess heterogeneity and pleiotropy. Gene Ontology enrichment analysis was performed to explore biological pathways. All analyses were conducted using R packages, including “TwoSampleMR,” “mr.raps,” and “MR-PRESSO.” Self-reported fatigue showed a significant causal association with any stroke, with an odds ratio of 1.43 (95% confidence interval: 1.04–1.96, *P* = .028) using the inverse-variance weighted method, supported by the weighted median method. However, no significant causal relationships were observed between fatigue and stroke subtypes (any ischemic stroke, large-artery atherosclerotic stroke, cardioembolic stroke, and small-vessel disease). Malnutrition was not causally associated with any type of stroke or its subtypes. Additionally, no significant causal effects of stroke or its subtypes on fatigue or malnutrition were detected. Sensitivity analyses confirmed the absence of heterogeneity or pleiotropy. Gene Ontology enrichment analysis revealed that biological pathways related to fatigue and any stroke primarily involved positive regulation of CREB transcription factor activity, synaptic function, and plasma membrane-related processes. Fatigue is a risk factor for any stroke, and its mediating biological pathways may include CREB transcription factor activity regulation, synaptic function, and plasma membrane-related processes. No causal relationships were identified between malnutrition and stroke or between stroke and fatigue.

## 1. Introduction

Stroke is a major global disease characterized by high incidence, disability, and mortality rates. Its occurrence is closely associated with a variety of factors, including genetics, environment, and lifestyle. Stroke not only imposes a heavy health and economic burden on patients and their families but also poses significant challenges to public health systems worldwide (https://www.world-stroke.org).^[1]^ Despite notable advancements in stroke prevention and treatment in recent years, the mechanisms underlying its pathogenesis and risk factors require further exploration. In addition to traditional risk factors such as hypertension, diabetes, and smoking, a growing body of research suggests that malnutrition and fatigue may play critical roles in the onset and progression of stroke.^[2,3]^

Malnutrition is a pathological condition resulting from insufficient energy or nutrient intake, poor absorption, or metabolic abnormalities. It is often accompanied by weakened immune function, muscle atrophy, and metabolic disorders.^[4]^ Malnutrition is also considered a potential risk factor for stroke, as deficiencies in nutrients such as vitamins, minerals, and antioxidants may lead to vascular endothelial dysfunction, exacerbated inflammatory responses, and increased risk of thrombosis.^[2]^ The prevalence of malnutrition is notably high among stroke patients, potentially due to dysphagia, reduced appetite, or increased metabolic demands associated with the disease.^[5]^ Meanwhile, fatigue, characterized by persistent tiredness and lack of energy, is a common symptom in various chronic conditions, including stroke.^[6]^ Post-stroke fatigue affects 30% to 70% of patients, significantly hindering recovery and quality of life.^[7]^ Furthermore, recent studies indicate that up to one-quarter of acute stroke patients experienced fatigue prior to the stroke event.^[8]^ Thus, fatigue may also serve as a precursor to stroke, as chronic fatigue can lead to reduced physical activity, metabolic disturbances, and increased psychological stress, indirectly elevating stroke risk.^[9]^ Although the associations between malnutrition, fatigue, and stroke have been widely reported, most evidence is derived from observational data, which are susceptible to confounding factors and reverse causality. Therefore, more robust causal inference methods are needed to validate these relationships.

Mendelian randomization (MR) is a genetic variant-based causal inference method that uses genetic variants associated with exposure factors (e.g., malnutrition or fatigue) as instrumental variables (IVs). This approach effectively mitigates confounding biases and reverse causality issues inherent in observational studies.^[10]^ In this study, we employed a bidirectional 2-sample MR analysis to systematically investigate the causal relationships between malnutrition, fatigue, and stroke. Specifically, we aimed to address 2 key scientific questions: whether genetically predicted malnutrition and fatigue are causally associated with an increased risk of stroke; and whether stroke, in turn, exerts a causal influence on the development of malnutrition and fatigue.

## 2. Materials and methods

### 2.1. Study design

**Figure 1.**
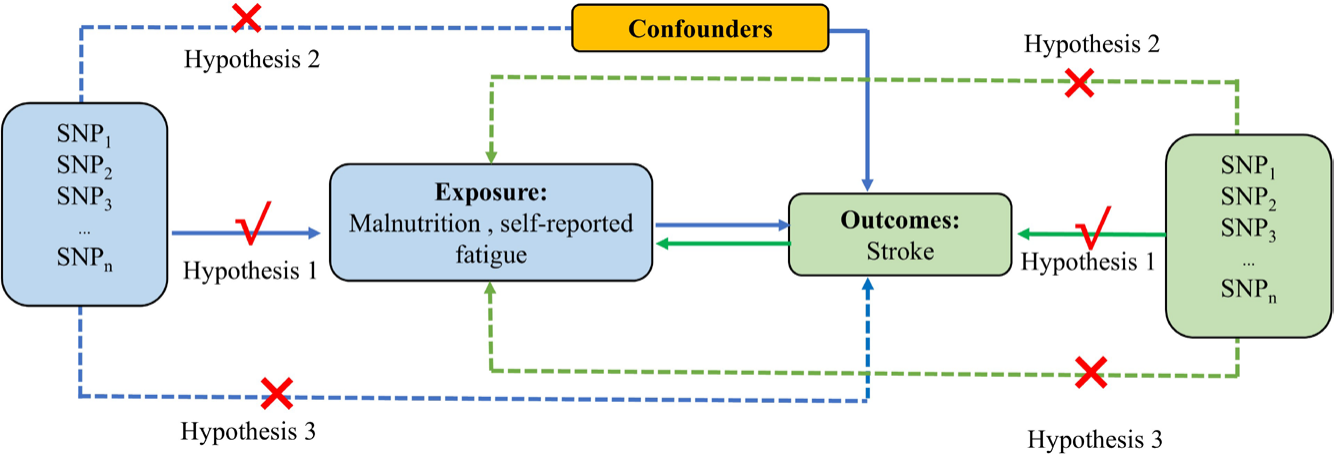
An overview of the MR study. MR = Mendelian randomization.

This study adheres to the Strengthening the Reporting of Mendelian Randomization Studies (STROBE-MR) guidelines^[11]^ and employs a 2-sample MR approach to investigate the causal relationships between malnutrition, fatigue, and the risk of stroke and its subtypes. The study flowchart is presented in Figure 1. This research is based on the following assumptions^[12,13]^: 1st, genetic variants are strongly associated with stroke; 2nd, genetic variants are independent of confounding factors; and 3rd, genetic variants influence stroke solely through the exposure factors (malnutrition and fatigue) and not via other pathways.

#### 2.1.1. Data collection

The data on malnutrition and fatigue used in this study were obtained from the Genome-Wide Association Study (GWAS) Catalog (https://www.ebi.ac.uk/gwas/).^[14,15]^ The stroke data were derived from publicly available summary statistics on genetic associations for stroke and its subtypes, provided by the MEGASTROKE consortium. This dataset included 40,585 stroke cases and 406,111 controls of European ancestry.^[16]^ Stroke was defined as rapidly developing clinical signs of focal (or global) neurological dysfunction lasting ≥ 24 hours or leading to death, with no apparent cause other than vascular origin. The GWAS data encompassed 34,217 ischemic stroke cases, which were further classified into 3 subtypes based on the Trial of Org 10172 in Acute Stroke Treatment criteria: large-artery atherosclerotic stroke (LAAS, 4373 cases), cardioembolic stroke (CEI, 7193 cases), and small-vessel disease (SVD, 5386 cases).^[17]^ These data are accessible through the IEU OpenGWAS database (https://gwas.mrcieu.ac.uk/). All data and materials used in this study are publicly available, with specific sources detailed in the text and Table 1. As the study relied on published research databases, no additional ethical approval or participant consent was required.

**Table 1:** Details of data sources included in the study.

GWAS ID	Phenotype	Data source	Population	Sample size (cases/controls)	PubMed ID
GCST90084864	Self-reported fatigue	UK Biobank	European	454,787	34662886
GCST90435738	Malnutrition	-	European	407,549	30104761
ebi-a-GCST005838	Any stroke	MEGASTROKE	European	40,585/406,111	29531354
ebi-a-GCST005843	Any ischemic stroke	MEGASTROKE	European	34 ,217/406,111	29531354
ebi-a-GCST006907	LAAS	MEGASTROKE	European	4373/406,111	29531354
ebi-a-GCST006909	SVD	MEGASTROKE	European	5386/192,662	29531354
ebi-a-GCST006910	CEI	MEGASTROKE	European	7193/406,111	29531354

Data summarized from GWAS databases from IEU OpenGWAS database (https://gwas.mrcieu.ac.uk/).

CEI = cardioembolic infarction, LAAS = large-artery atherosclerotic stroke, SVD = small-vessel disease.

### 2.2. Instrument variants selection

The selection of our IVs followed stringent criteria to ensure that the chosen IVs satisfy the 3 core assumptions of MR, thereby guaranteeing the reliability and accuracy of the MR analysis. First, we selected single nucleotide polymorphisms (SNPs) that exhibited a statistically significant association with the exposure factors using a genome-wide significance threshold (*P* < 5 × 10^8^). Second, we applied a clumping procedure with an *R*² threshold of < 0.001 and a genetic distance of > 10,000 kb to exclude SNPs in linkage disequilibrium. Third, to assess the strength of the IVs, we calculated the F-statistic for each SNP using the formula *F* = *R*² (*N* − *K* − 1)/[*K*(1 − *R*²)], where *N* is the sample size and *K* is the number of IVs. IVs with an *F*-statistic <10 were considered weak instruments and were excluded from further analysis.^[18–20]^ Finally, SNPs with palindromic alleles and those not present in the outcome GWAS were also excluded (Tables S1 and S2, Supplemental Digital Content, https://links.lww.com/MD/R49).

### 2.3. Statistical analysis

In the MR analysis, the inverse-variance weighted (IVW) method was employed as our primary analytical approach. This method combines Wald ratio estimates from multiple SNPs through meta-analysis to assess the overall effect of the exposure on the outcome. It is a widely used and statistically robust approach.^[21]^ Additionally, we utilized the following complementary methods to evaluate the robustness and reliability of the results: the weighted median method, which provides consistent estimates and reliable conclusions when more than half of the SNPs are valid IVs^[22]^; MR Egger regression, which estimates causal effects in the presence of horizontal pleiotropy through weighted linear regression^[12]^; the simple mode method, which identifies potential causal relationships by determining the most common causal effect estimate; and the weighted mode method, which assigns different weights to various causal effect estimates to identify the most likely causal relationship. Collectively, these comprehensive methods were applied to investigate the causal relationships between malnutrition, fatigue, and stroke (including its subtypes), ensuring the robustness and reliability of the findings.

### 2.4. Sensitivity analysis

To ensure the robustness of our findings, we conducted sensitivity analyses using multiple approaches. First, Cochran *Q* test was performed to evaluate heterogeneity among SNP-specific estimates. A *P*-value < .05 indicated significant heterogeneity, prompting the use of the fixed-effects model in the IVW method for MR analysis; otherwise, the random-effects model was applied. Second, the MR Egger intercept test was utilized to assess horizontal pleiotropy among SNPs. Additionally, the MR-PRESSO test was employed to further investigate potential pleiotropic effects. Third, funnel plots were generated to visually inspect potential pleiotropy, while scatter plots were created to illustrate the associations between exposures and outcomes. Fourth, a leave-one-out sensitivity analysis was conducted to determine whether the results were influenced by any individual SNP. For outcome measures, odds ratios with 95% confidence intervals were calculated to estimate the magnitude of causal relationships. All statistical analyses were performed using *R* software (version 4.4.0; R Foundation for Statistical Computing, Vienna, Austria) with the “TwoSampleMR,” “mr.raps,” and “MR-PRESSO” packages.

### 2.5. Enrichment analysis

To explore the biological pathways and mechanisms associated with risk genes linked to malnutrition, fatigue, stroke, and its subtypes, Gene Ontology enrichment analysis was conducted. This analysis assessed the enrichment of genes across 3 categories: cellular components, biological processes, and molecular functions.

## 3. Results

### 3.1. Causal effects of malnutrition on stroke

**Figure 2.**
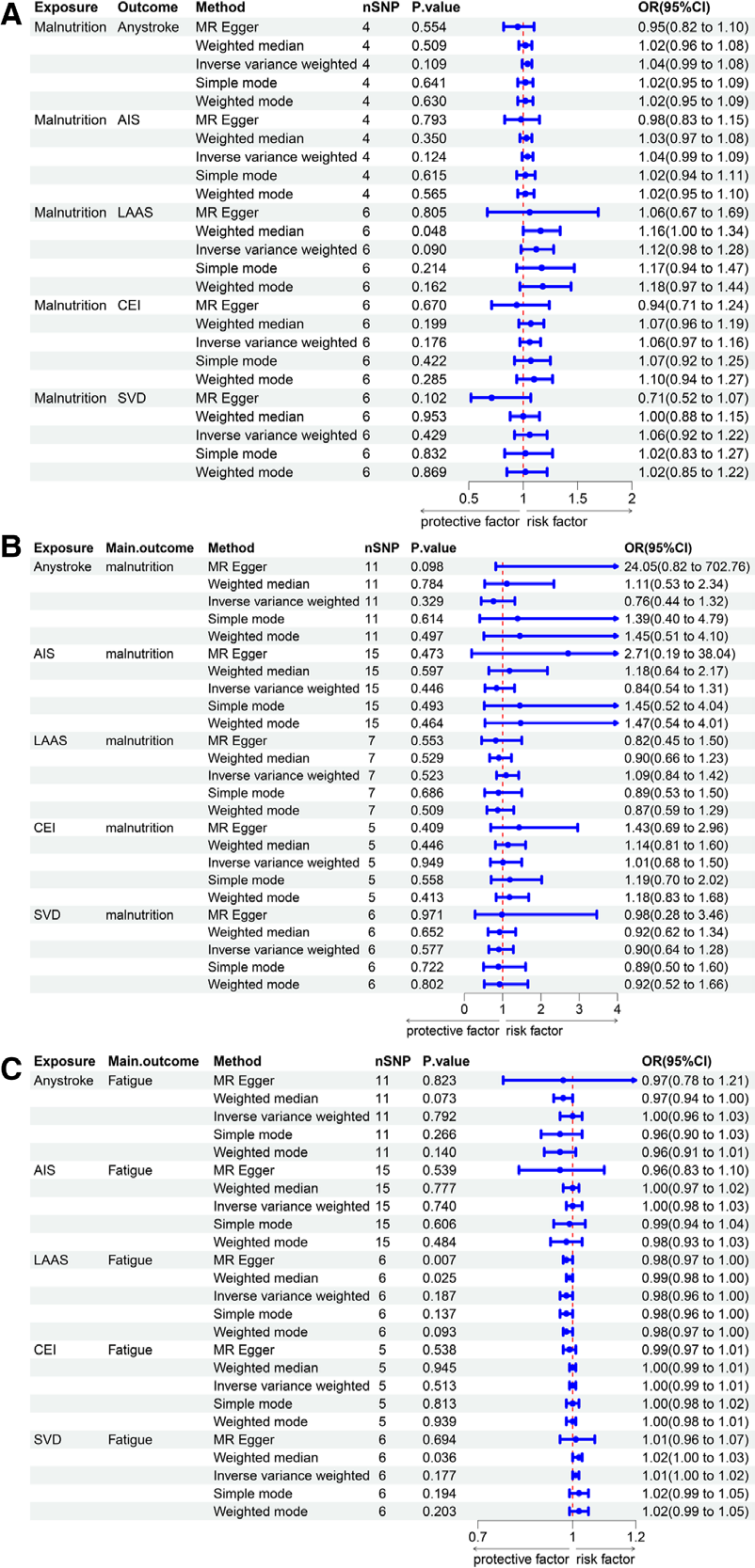
Forest plots illustrating the results of MR analyses: (A) the association between malnutrition and stroke risk, (B) the association between stroke and malnutrition, and (C) the association between stroke and fatigue. MR = Mendelian randomization.

As shown in Figure 2A, the results of the MR analysis indicate no causal effects of malnutrition on all types of stroke. The relevant SNPs are detailed in Table 2 and Figure S1C, F, I, L, and O, Supplemental Digital Content, https://links.lww.com/MD/R49. Scatter plots and funnel plots are also provided in Figure S1A, B, D, E, G, H, J, K, M, and N, Supplemental Digital Content, https://links.lww.com/MD/R49. Furthermore, no heterogeneity was observed based on Cochran *Q* test (*P*-values > .05) (Table 3). The MR Egger regression method showed no evidence of pleiotropy (Table 3). Similarly, the MR-PRESSO analysis demonstrated no significant influence on the conclusions.

**Table 2:** Statistic of sample size and instrumental variables.

Exposure		Outcome		Instrumental variables (nSNPs)
Trait	Sample size (cases/controls)	Trait	Sample size (cases/controls)
Malnutrition	407,549	Any stroke	40,585/406,111	4
Malnutrition	407,549	any ischemic stroke	34 ,217/406,111	4
Malnutrition	407,549	LAAS	4373/406,111	6
Malnutrition	407,549	CEI	7193/406,111	6
Malnutrition	407,549	SVD	5386/192,662	6
Fatigue	454,787	Any stroke	40,585/406,111	34
Fatigue	454,787	any ischemic stroke	34 ,217/406,111	34
Fatigue	454,787	LAAS	4373/406,111	35
Fatigue	454,787	CEI	7193/406,111	35
Fatigue	454,787	SVD	5386/192,662	35
Any stroke	40,585/406,111	Malnutrition	407,549	11
any ischemic stroke	34 ,217/406,111	Malnutrition	407,549	15
LAAS	4373/406,111	Malnutrition	407,549	7
CEI	7193/406,111	Malnutrition	407,549	5
SVD	5386/192,662	Malnutrition	407,549	6
Any stroke	40,585/406,111	Fatigue	454,787	11
any ischemic stroke	34 ,217/406,111	Fatigue	454,787	15
LAAS	4373/406,111	Fatigue	454,787	6
CEI	7193/406,111	Fatigue	454,787	5
SVD	5386/192,662	Fatigue	454,787	6

CEI = cardioembolic infarction, LAAS = large-artery atherosclerotic stroke, SNP = single nucleotide polymorphism, SVD = small-vessel disease.

**Table 3:** Pleiotropy, heterogeneity, and MR-PRESSO assessment.

		Pleiotropy			Heterogeneity				Heterogeneity			
Exposure	Outcome	Intercept	SE	*P*-value	Method	*Q*	df	*Q* value	Method	*Q*	df	*Q* value
Malnutrition	Any stroke	0.043	0.035	.347	MR Egger	0.837	2	0.658	IVW	2.326	3	0.508
Malnutrition	Any ischemic stroke	0.029	0.037	.516	MR Egger	0.786	2	0.675	IVW	1.398	3	0.706
Malnutrition	LAAS	0.027	0.114	.826	MR Egger	7.648	4	0.105	IVW	7.754	5	0.170
Malnutrition	CEI	0.063	0.068	.406	MR Egger	0.600	4	0.963	IVW	1.459	5	0.918
Malnutrition	SVD	0.203	0.078	.060	MR Egger	2.787	4	0.594	IVW	9.558	5	0.089
Fatigue	Any stroke	0.013	0.011	.232	MR Egger	39.788	32	0.162	IVW	41.635	33	0.144
Fatigue	Any ischemic stroke	0.011	0.011	.314	MR Egger	39.547	32	0.169	IVW	40.842	33	0.164
Fatigue	LAAS	0.022	0.030	.478	MR Egger	44.901	33	0.081	IVW	45.601	34	0.088
Fatigue	CEI	0.009	0.022	.689	MR Egger	39.331	33	0.207	IVW	39.526	34	0.237
Fatigue	SVD	-0.032	0.027	.245	MR Egger	40.364	33	0.177	IVW	42.075	34	0.161
Any stroke	Malnutrition	-0.196	0.097	.073	MR Egger	6.674	9	0.671	IVW	10.798	10	0.373
Any ischemic stroke	Malnutrition	-0.068	0.077	.395	MR Egger	10.891	13	0.620	IVW	11.664	14	0.633
LAAS	Malnutrition	0.081	0.079	.355	MR Egger	6.592	5	0.253	IVW	7.958	6	0.241
CEI	Malnutrition	-0.079	0.072	.356	MR Egger	6.471	3	0.091	IVW	9.025	4	0.060
SVD	Malnutrition	-0.014	0.118	.909	MR Egger	7.343	4	0.119	IVW	7.371	5	0.194
Any stroke	Fatigue	0.001	0.006	.851	MR Egger	23.179	9	0.006	IVW	23.275	10	0.010
Any ischemic stroke	Fatigue	0.003	0.004	.499	MR Egger	22.667	13	0.046	IVW	23.512	14	0.052
LAAS	Fatigue	0.002	0.003	.490	MR Egger	3.970	4	0.410	IVW	4.548	5	0.474
CEI	Fatigue	0.002	0.002	.308	MR Egger	2.467	3	0.481	IVW	3.964	4	0.411
SVD	Fatigue	0.000	0.005	.960	MR Egger	7.733	4	0.102	IVW	7.739	5	0.171

CEI = cardioembolic infarction, LAAS = large-artery atherosclerotic stroke, MR = Mendelian randomization, SVD = small-vessel disease.

### 3.2. Causal effects of self-reported fatigue on stroke

**Figure 3.**
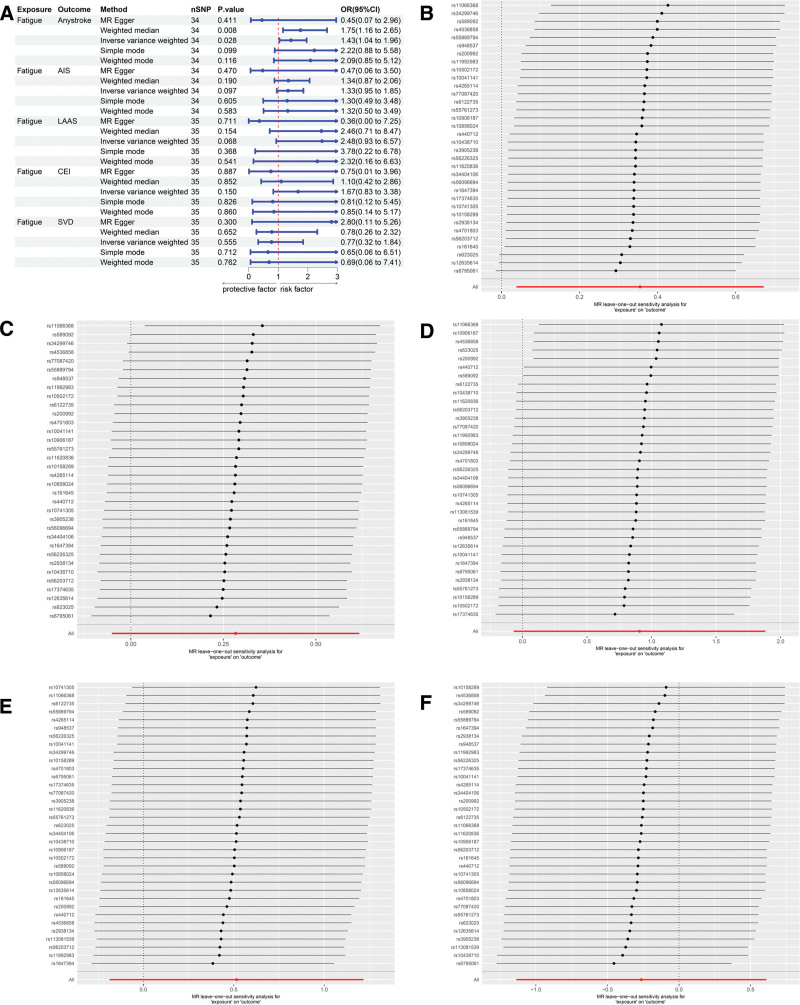
Forest plots from Mendelian randomization analyses: (A) the association between fatigue and stroke risk, and leave-one-out sensitivity analyses for the associations between fatigue and any stroke (B), any ischemic stroke (C), LAAS (D), CEI (E), and SVD (F). CEI = cardioembolic stroke, LAAS = large-artery atherosclerotic stroke, SVD = small-vessel disease.

We also evaluated the causal relationship between self-reported fatigue and stroke. The MR analysis results indicated a causal relationship between self-reported fatigue and any stroke, with the IVW method yielding an odds ratios of 1.43 (95% confidence interval: 1.04–1.96, *P* = .028). This conclusion was further supported by the weighted median method (Fig. 3A and B). Cochran *Q* test revealed no heterogeneity or pleiotropy (Table 3). Additionally, no significant causal relationships were observed between self-reported fatigue and any ischemic stroke, LAAS, CEI, or SVD (Figure 3A, C, D, E, and F). Finally, scatter plots and funnel plots illustrating the causal relationships between fatigue and various stroke subtypes were generated (Figure S2A–J, Supplemental Digital Content, https://links.lww.com/MD/R49).

**Figure 4.**
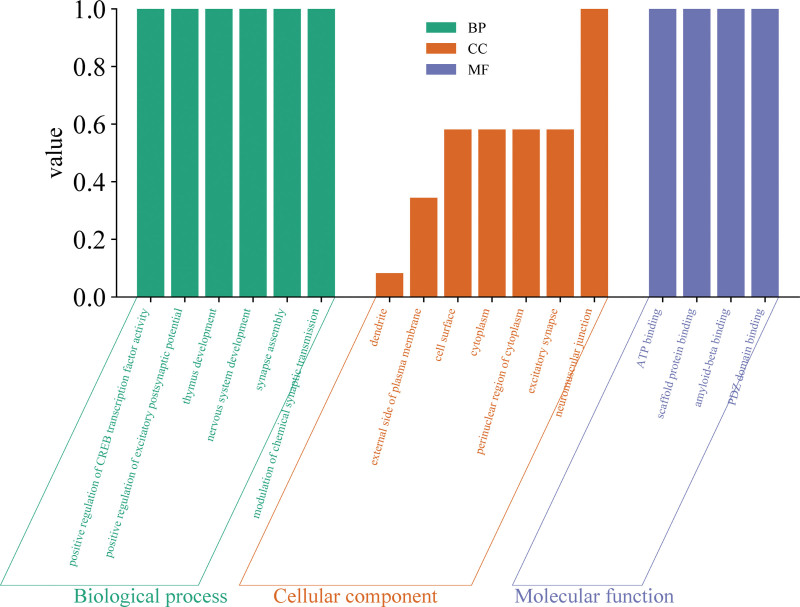
Bar plot of GO enrichment analysis illustrating the biological pathways associated with the causal relationship between fatigue and all-cause stroke (any stroke). GO = Gene Ontology.

As shown in Figure 4 and Table S3, Supplemental Digital Content, https://links.lww.com/MD/R49, Gene Ontology enrichment analysis revealed that, in terms of biological processes, the findings were primarily associated with “positive regulation of CREB transcription factor activity,” “positive regulation of excitatory postsynaptic potential,” and “thymus development.” For cellular components, the results were mainly linked to “dendrite,” “external side of plasma membrane,” “cell surface,” “cytoplasm,” “perinuclear region of cytoplasm,” and “excitatory synapse.” In terms of molecular functions, the findings were predominantly associated with “ATP binding,” “scaffold protein binding,” “amyloid-beta binding,” and “PDZ domain binding.”

### 3.3. Causal effects of stroke on malnutrition

Furthermore, we evaluated the causal effects of various types of stroke (any stroke, any ischemic stroke, LAAS, CEI, SVD) on malnutrition. No direct causal relationships were observed between any type of stroke and malnutrition (Fig. 2B). Additionally, Cochran *Q* test indicated no heterogeneity or pleiotropy in our analyses (Table 3). Finally, scatter plots, funnel plots, and leave-one-out sensitivity analysis results illustrating the causal relationships between different types of stroke and malnutrition were generated (Figure S3A–O, Supplemental Digital Content, https://links.lww.com/MD/R49).

### 3.4. Causal effects of stroke on self-reported fatigue

Lastly, we assessed the causal effects of various types of stroke (any stroke, any ischemic stroke, LAAS, CEI, SVD) on self-reported fatigue. No direct causal relationships were found between any type of stroke and fatigue (Fig. 2B). Cochran *Q* test revealed no heterogeneity or pleiotropy in our analysis results (Table 3). Similarly, scatter plots, funnel plots, and leave-one-out sensitivity analysis results demonstrating the causal relationships between different types of stroke and fatigue were provided (Figure S4A–O, Supplemental Digital Content, https://links.lww.com/MD/R49).

## 4. Discussion

This study is the 1st to explore the causal relationships between malnutrition, self-reported fatigue, and different types of stroke. Through MR analysis, we concluded that self-reported fatigue may increase the risk of all-cause stroke (any stroke), which is consistent with findings from previous clinical studies. However, we found no convincing evidence to support a causal relationship between malnutrition and stroke, nor did we identify causal relationships between fatigue and specific ischemic stroke subtypes, including LAAS, CEI, and SVD.

The association between self-reported fatigue and the risk of all-cause stroke has been reported in previous epidemiological observations.^[23]^ This finding suggests that fatigue may be an important predictor of stroke. Fatigue, as a multidimensional syndrome, is often characterized by reduced physiological function, weakened muscle strength, and abnormal energy metabolism, which may increase stroke risk through various pathways.^[24]^ For example, patients with fatigue often exhibit chronic inflammation, endothelial dysfunction, and autonomic dysregulation, which may directly or indirectly promote thrombosis or vascular injury, thereby increasing the likelihood of stroke.^[25,26]^ Additionally, fatigue is frequently associated with comorbidities such as hypertension, diabetes, and atrial fibrillation, which are themselves significant risk factors for stroke.^[27]^ Therefore, fatigue may synergistically increase the overall risk of stroke through multiple mechanisms. Based on these findings, we have sufficient reason to conclude that fatigue increases the risk of all-cause stroke.

Regarding the causal relationship between fatigue and stroke subtypes (any ischemic stroke, LAAS, CEI, SVD), this study did not reach a definitive conclusion. This may be related to the heterogeneity of stroke subtypes.^[17]^ Different types of stroke have distinct etiologies and pathophysiological mechanisms, and fatigue may have a more pronounced impact on certain subtypes while exerting weaker effects on others.^[28]^ For instance, any ischemic stroke and LAAS are closely associated with atherosclerosis, while CEI is linked to cardioembolism, and SVD primarily involves pathological changes in small vessels.^[29]^ Fatigue may influence the progression of atherosclerosis or cardiac function, thereby having a more significant impact on certain stroke subtypes, while its effects on others may be relatively limited.^[30,31]^ Therefore, future studies may need to conduct more detailed analyses of specific stroke subtypes to uncover potential associations between fatigue and particular types of stroke.

However, this study did not find a significant causal relationship between malnutrition and stroke. This result may be related to the definition and measurement methods of malnutrition. Malnutrition is typically assessed using indicators such as body mass index, serum albumin levels, or nutritional assessment tools, but these metrics may not fully capture an individual’s nutritional status.^[32,33]^ Furthermore, the relationship between malnutrition and stroke may be influenced by various confounding factors, such as socioeconomic status, lifestyle, and chronic diseases.^[34,35]^ Future research may need to employ more precise nutritional assessment methods and incorporate multidimensional data to further explore the potential association between malnutrition and stroke.^[36]^

Due to the limitations of observational studies and the challenges of implementing prospective clinical research, this study utilized SNPs specifically related to exposures or outcomes as IVs to assess the causal relationships between malnutrition, fatigue, and stroke risk. The results indicated a causal relationship between fatigue and all-cause stroke but found no associations with specific stroke subtypes. By employing MR, this study revealed a potential causal link between self-reported fatigue and all-cause stroke, providing new insights for stroke prevention and management. The MR method effectively reduced confounding bias, and the use of large-scale GWAS data ensured the reliability and statistical power of the findings. However, the association between malnutrition and stroke was not confirmed, which may be attributed to the limitations of nutritional assessment methods. Future studies should adopt more comprehensive nutritional evaluation tools.

Although this study is the 1st to explore the relationship between fatigue and stroke subtypes (e.g., any ischemic stroke, LAAS, CEI, SVD), no significant associations were identified due to sample size limitations and the heterogeneity of stroke subtypes. Additionally, the pleiotropy of genetic IVs and population specificity may affect the generalizability of the results. Future research should expand sample sizes, optimize IVs, and validate findings across diverse populations to further elucidate the complex mechanisms linking fatigue and malnutrition to stroke, providing a scientific basis for precise prevention and treatment.

## 5. Conclusion

In summary, this study, through MR analysis, revealed a potential causal relationship between self-reported fatigue and all-cause stroke (any stroke). However, no association was found between malnutrition and stroke, nor were significant relationships identified between fatigue and specific stroke subtypes. These findings provide new insights for stroke prevention and management, suggesting that the assessment and intervention of fatigue should be emphasized in clinical practice to reduce the risk of stroke. Nevertheless, future research is needed to further explore the complex relationships between malnutrition, fatigue, and stroke, particularly the specific mechanisms underlying different stroke subtypes. Such efforts will contribute to a more robust scientific foundation for the precise prevention and treatment of stroke.

## Acknowledgments

All datasets utilized in the present investigation were sourced from publicly accessible databases and collaborative consortia. The authors extend their gratitude to these entities for facilitating access to the data, which was instrumental in the execution of this research.

## Author contributions

**Conceptualization:** Min Luo, Minyue Sun.

**Data curation:** Min Luo, Shiyu Wen, Huiping Jiang, Yu Lei.

**Project administration:** Minyue Sun.

**Visualization:** Shiyu Wen, Huiping Jiang, Yu Lei.

**Writing – original draft:** Min Luo.

**Writing – review & editing:** Minyue Sun.

## Supplementary Material


