# Supplementary material for: Assessing the bidirectional causal relationship between malnutrition, self-reported fatigue, and stroke: A 2-sample Mendelian randomization study
Source: Medicine (Baltimore). 2026 Jan 2;105(1):e46894. doi: 10.1097/MD.0000000000046894 (PMC12778199; doi:10.1097/MD.0000000000046894)

**Table S1. Characteristics of selected single nucleotide polymorphisms concerning the causal effect of malnutrition, fatigue on stroke**

| Exposure | Outcome | SNP | effect_allele.exposure | other_allele.exposure | pval.exposure | beta.exposure | se.exposure |
|---|---|---|---|---|---|---|---|
| Malnutrition | Any stroke | rs115854809 | T | C | 3.48E-06 | 0.596 | 0.128 |
| Malnutrition | Any stroke | rs143001311 | T | C | 4.51E-07 | 0.684 | 0.136 |
| Malnutrition | Any stroke | rs62175763 | T | A | 2.88E-06 | 0.266 | 0.0567 |
| Malnutrition | Any stroke | rs72666215 | C | A | 2.87E-06 | 0.4 | 0.0855 |
| Malnutrition | Any stroke | rs76765288 | C | T | 1.87E-06 | 0.33 | 0.0693 |
| Malnutrition | Any ischemic stroke | rs115854809 | T | C | 3.48E-06 | 0.596 | 0.128 |
| Malnutrition | Any ischemic stroke | rs143001311 | T | C | 4.51E-07 | 0.684 | 0.136 |
| Malnutrition | Any ischemic stroke | rs62175763 | T | A | 2.88E-06 | 0.266 | 0.0567 |
| Malnutrition | Any ischemic stroke | rs72666215 | C | A | 2.87E-06 | 0.4 | 0.0855 |
| Malnutrition | Any ischemic stroke | rs76765288 | C | T | 1.87E-06 | 0.33 | 0.0693 |
| Malnutrition | LAAS | rs115854809 | T | C | 3.48E-06 | 0.596 | 0.128 |
| Malnutrition | LAAS | rs116036397 | G | A | 3.69E-06 | 0.681 | 0.147 |
| Malnutrition | LAAS | rs143001311 | T | C | 4.51E-07 | 0.684 | 0.136 |
| Malnutrition | LAAS | rs62175763 | T | A | 2.88E-06 | 0.266 | 0.0567 |
| Malnutrition | LAAS | rs72664333 | A | G | 4.68E-06 | 0.808 | 0.177 |
| Malnutrition | LAAS | rs72666215 | C | A | 2.87E-06 | 0.4 | 0.0855 |
| Malnutrition | LAAS | rs76765288 | C | T | 1.87E-06 | 0.33 | 0.0693 |
| Malnutrition | CEI | rs115854809 | T | C | 3.48E-06 | 0.596 | 0.128 |
| Malnutrition | CEI | rs116036397 | G | A | 3.69E-06 | 0.681 | 0.147 |
| Malnutrition | CEI | rs143001311 | T | C | 4.51E-07 | 0.684 | 0.136 |
| Malnutrition | CEI | rs62175763 | T | A | 2.88E-06 | 0.266 | 0.0567 |
| Malnutrition | CEI | rs72664333 | A | G | 4.68E-06 | 0.808 | 0.177 |
| Malnutrition | CEI | rs72666215 | C | A | 2.87E-06 | 0.4 | 0.0855 |
| Malnutrition | CEI | rs76765288 | C | T | 1.87E-06 | 0.33 | 0.0693 |

| | | | | | | | |
|---|---|---|---|---|---|---|---|
| Malnutrition | SVD | rs115854809 | T | C | 3.48E-06 | 0.596 | 0.128 |
| Malnutrition | SVD | rs116036397 | G | A | 3.69E-06 | 0.681 | 0.147 |
| Malnutrition | SVD | rs143001311 | T | C | 4.51E-07 | 0.684 | 0.136 |
| Malnutrition | SVD | rs62175763 | T | A | 2.88E-06 | 0.266 | 0.0567 |
| Malnutrition | SVD | rs72664333 | A | G | 4.68E-06 | 0.808 | 0.177 |
| Malnutrition | SVD | rs72666215 | C | A | 2.87E-06 | 0.4 | 0.0855 |
| Malnutrition | SVD | rs76765288 | C | T | 1.87E-06 | 0.33 | 0.0693 |
| Fatigue | Any stroke | rs10041141 | C | T | 1.50E-08 | -0.00993469 | 0.00175377 |
| Fatigue | Any stroke | rs10158289 | G | A | 3.00E-08 | -0.00972082 | 0.00175356 |
| Fatigue | Any stroke | rs10438710 | G | A | 4.80E-13 | -0.0157706 | 0.00218063 |
| Fatigue | Any stroke | rs10502172 | T | C | 4.10E-08 | -0.00956779 | 0.00174386 |
| Fatigue | Any stroke | rs10741305 | C | T | 3.90E-08 | 0.0096179 | 0.00174982 |
| Fatigue | Any stroke | rs10858024 | A | C | 3.40E-08 | 0.00977954 | 0.00177199 |
| Fatigue | Any stroke | rs10906187 | G | A | 6.00E-10 | 0.0111693 | 0.00180391 |
| Fatigue | Any stroke | rs11066368 | A | G | 5.20E-09 | 0.0105112 | 0.00179997 |
| Fatigue | Any stroke | rs11620836 | T | C | 9.20E-10 | -0.0124167 | 0.00202811 |
| Fatigue | Any stroke | rs11992983 | A | G | 1.70E-08 | 0.00994216 | 0.00176162 |
| Fatigue | Any stroke | rs12635614 | A | G | 3.90E-08 | 0.00959086 | 0.00174511 |
| Fatigue | Any stroke | rs139498 | C | G | 1.10E-08 | -0.0100756 | 0.00176391 |
| Fatigue | Any stroke | rs161645 | G | A | 2.30E-08 | -0.0104122 | 0.00186423 |
| Fatigue | Any stroke | rs1647394 | T | C | 4.10E-08 | 0.00970963 | 0.00176921 |
| Fatigue | Any stroke | rs17374635 | C | T | 4.40E-08 | -0.0103017 | 0.00188205 |
| Fatigue | Any stroke | rs200992 | G | A | 2.20E-08 | -0.0151129 | 0.00270229 |
| Fatigue | Any stroke | rs2186710 | G | C | 1.40E-08 | 0.0101419 | 0.00178599 |
| Fatigue | Any stroke | rs2938134 | A | C | 4.90E-08 | -0.010173 | 0.00186554 |
| Fatigue | Any stroke | rs34299746 | T | G | 2.30E-10 | -0.0112145 | 0.00176963 |

| | | | | | | | |
|---|---|---|---|---|---|---|---|
| Fatigue | Any stroke | rs34404106 | G | A | 4.80E-08 | -0.0104331 | 0.00191174 |
| Fatigue | Any stroke | rs3905238 | G | A | 6.60E-09 | -0.0103144 | 0.0017781 |
| Fatigue | Any stroke | rs426444 | A | T | 1.20E-08 | -0.010648 | 0.00186685 |
| Fatigue | Any stroke | rs4265114 | C | A | 3.10E-08 | 0.00965894 | 0.00174509 |
| Fatigue | Any stroke | rs440712 | A | G | 3.30E-08 | 0.010018 | 0.00181335 |
| Fatigue | Any stroke | rs4536858 | G | A | 1.80E-09 | -0.0108444 | 0.00180229 |
| Fatigue | Any stroke | rs4701803 | T | C | 2.30E-08 | -0.00985094 | 0.00176256 |
| Fatigue | Any stroke | rs55761273 | G | A | 4.70E-08 | 0.0135133 | 0.00247427 |
| Fatigue | Any stroke | rs55889794 | C | T | 1.70E-08 | 0.0294965 | 0.00523235 |
| Fatigue | Any stroke | rs56096694 | T | C | 3.20E-08 | 0.0102745 | 0.00185768 |
| Fatigue | Any stroke | rs56203712 | G | A | 3.60E-08 | -0.0116093 | 0.00210732 |
| Fatigue | Any stroke | rs56226325 | T | C | 3.50E-08 | -0.0132474 | 0.00240298 |
| Fatigue | Any stroke | rs589092 | G | A | 1.00E-08 | -0.0100074 | 0.00174664 |
| Fatigue | Any stroke | rs6122735 | T | C | 1.80E-09 | 0.010716 | 0.00178282 |
| Fatigue | Any stroke | rs62097947 | T | A | 3.40E-08 | 0.00986865 | 0.00178718 |
| Fatigue | Any stroke | rs623025 | C | T | 1.70E-08 | 0.011296 | 0.0020034 |
| Fatigue | Any stroke | rs6795061 | C | T | 2.00E-08 | 0.0109787 | 0.00195759 |
| Fatigue | Any stroke | rs7035657 | A | T | 4.90E-08 | 0.013833 | 0.00253576 |
| Fatigue | Any stroke | rs77087420 | G | A | 5.40E-09 | -0.0223936 | 0.00383847 |
| Fatigue | Any stroke | rs948537 | T | C | 2.30E-08 | 0.0117334 | 0.00209935 |
| Fatigue | Any ischemic stroke | rs10041141 | T | C | 1.50E-08 | -0.00993469 | 0.00175377 |
| Fatigue | Any ischemic stroke | rs10158289 | A | G | 3.00E-08 | -0.00972082 | 0.00175356 |
| Fatigue | Any ischemic stroke | rs10438710 | A | G | 4.80E-13 | -0.0157706 | 0.00218063 |
| Fatigue | Any ischemic stroke | rs10502172 | C | T | 4.10E-08 | -0.00956779 | 0.00174386 |
| Fatigue | Any ischemic stroke | rs10741305 | T | C | 3.90E-08 | 0.0096179 | 0.00174982 |
| Fatigue | Any ischemic stroke | rs10858024 | C | A | 3.40E-08 | 0.00977954 | 0.00177199 |

| | | | | | | | |
|---|---|---|---|---|---|---|---|
| Fatigue | Any ischemic stroke | rs10906187 | A | G | 6.00E-10 | 0.0111693 | 0.00180391 |
| Fatigue | Any ischemic stroke | rs11066368 | G | A | 5.20E-09 | 0.0105112 | 0.00179997 |
| Fatigue | Any ischemic stroke | rs11620836 | C | T | 9.20E-10 | -0.0124167 | 0.00202811 |
| Fatigue | Any ischemic stroke | rs11992983 | G | A | 1.70E-08 | 0.00994216 | 0.00176162 |
| Fatigue | Any ischemic stroke | rs12635614 | G | A | 3.90E-08 | 0.00959086 | 0.00174511 |
| Fatigue | Any ischemic stroke | rs139498 | G | C | 1.10E-08 | -0.0100756 | 0.00176391 |
| Fatigue | Any ischemic stroke | rs161645 | A | G | 2.30E-08 | -0.0104122 | 0.00186423 |
| Fatigue | Any ischemic stroke | rs1647394 | C | T | 4.10E-08 | 0.00970963 | 0.00176921 |
| Fatigue | Any ischemic stroke | rs17374635 | T | C | 4.40E-08 | -0.0103017 | 0.00188205 |
| Fatigue | Any ischemic stroke | rs200992 | A | G | 2.20E-08 | -0.0151129 | 0.00270229 |
| Fatigue | Any ischemic stroke | rs2186710 | C | G | 1.40E-08 | 0.0101419 | 0.00178599 |
| Fatigue | Any ischemic stroke | rs2938134 | C | A | 4.90E-08 | -0.010173 | 0.00186554 |
| Fatigue | Any ischemic stroke | rs34299746 | G | T | 2.30E-10 | -0.0112145 | 0.00176963 |
| Fatigue | Any ischemic stroke | rs34404106 | A | G | 4.80E-08 | -0.0104331 | 0.00191174 |
| Fatigue | Any ischemic stroke | rs3905238 | A | G | 6.60E-09 | -0.0103144 | 0.0017781 |
| Fatigue | Any ischemic stroke | rs426444 | T | A | 1.20E-08 | -0.010648 | 0.00186685 |
| Fatigue | Any ischemic stroke | rs4265114 | A | C | 3.10E-08 | 0.00965894 | 0.00174509 |
| Fatigue | Any ischemic stroke | rs440712 | G | A | 3.30E-08 | 0.010018 | 0.00181335 |
| Fatigue | Any ischemic stroke | rs4536858 | A | G | 1.80E-09 | -0.0108444 | 0.00180229 |
| Fatigue | Any ischemic stroke | rs4701803 | C | T | 2.30E-08 | -0.00985094 | 0.00176256 |
| Fatigue | Any ischemic stroke | rs55761273 | A | G | 4.70E-08 | 0.0135133 | 0.00247427 |
| Fatigue | Any ischemic stroke | rs55889794 | T | C | 1.70E-08 | 0.0294965 | 0.00523235 |
| Fatigue | Any ischemic stroke | rs56096694 | C | T | 3.20E-08 | 0.0102745 | 0.00185768 |
| Fatigue | Any ischemic stroke | rs56203712 | A | G | 3.60E-08 | -0.0116093 | 0.00210732 |
| Fatigue | Any ischemic stroke | rs56226325 | C | T | 3.50E-08 | -0.0132474 | 0.00240298 |
| Fatigue | Any ischemic stroke | rs589092 | A | G | 1.00E-08 | -0.0100074 | 0.00174664 |

| | | | | | | | |
|---|---|---|---|---|---|---|---|
| Fatigue | Any ischemic stroke | rs6122735 | C | T | 1.80E-09 | 0.010716 | 0.00178282 |
| Fatigue | Any ischemic stroke | rs62097947 | A | T | 3.40E-08 | 0.00986865 | 0.00178718 |
| Fatigue | Any ischemic stroke | rs623025 | T | C | 1.70E-08 | 0.011296 | 0.0020034 |
| Fatigue | Any ischemic stroke | rs6795061 | T | C | 2.00E-08 | 0.0109787 | 0.00195759 |
| Fatigue | Any ischemic stroke | rs7035657 | T | A | 4.90E-08 | 0.013833 | 0.00253576 |
| Fatigue | Any ischemic stroke | rs77087420 | A | G | 5.40E-09 | -0.0223936 | 0.00383847 |
| Fatigue | Any ischemic stroke | rs948537 | C | T | 2.30E-08 | 0.0117334 | 0.00209935 |
| Fatigue | LAAS | rs10041141 | C | T | 1.50E-08 | -0.00993469 | 0.00175377 |
| Fatigue | LAAS | rs10158289 | G | A | 3.00E-08 | -0.00972082 | 0.00175356 |
| Fatigue | LAAS | rs10438710 | G | A | 4.80E-13 | -0.0157706 | 0.00218063 |
| Fatigue | LAAS | rs10502172 | T | C | 4.10E-08 | -0.00956779 | 0.00174386 |
| Fatigue | LAAS | rs10741305 | C | T | 3.90E-08 | 0.0096179 | 0.00174982 |
| Fatigue | LAAS | rs10858024 | A | C | 3.40E-08 | 0.00977954 | 0.00177199 |
| Fatigue | LAAS | rs10906187 | G | A | 6.00E-10 | 0.0111693 | 0.00180391 |
| Fatigue | LAAS | rs11066368 | A | G | 5.20E-09 | 0.0105112 | 0.00179997 |
| Fatigue | LAAS | rs113081539 | T | C | 2.50E-09 | 0.0202856 | 0.00340099 |
| Fatigue | LAAS | rs11620836 | T | C | 9.20E-10 | -0.0124167 | 0.00202811 |
| Fatigue | LAAS | rs11992983 | A | G | 1.70E-08 | 0.00994216 | 0.00176162 |
| Fatigue | LAAS | rs12635614 | A | G | 3.90E-08 | 0.00959086 | 0.00174511 |
| Fatigue | LAAS | rs139498 | C | G | 1.10E-08 | -0.0100756 | 0.00176391 |
| Fatigue | LAAS | rs161645 | G | A | 2.30E-08 | -0.0104122 | 0.00186423 |
| Fatigue | LAAS | rs1647394 | T | C | 4.10E-08 | 0.00970963 | 0.00176921 |
| Fatigue | LAAS | rs17374635 | C | T | 4.40E-08 | -0.0103017 | 0.00188205 |
| Fatigue | LAAS | rs200992 | G | A | 2.20E-08 | -0.0151129 | 0.00270229 |
| Fatigue | LAAS | rs2186710 | G | C | 1.40E-08 | 0.0101419 | 0.00178599 |
| Fatigue | LAAS | rs2938134 | A | C | 4.90E-08 | -0.010173 | 0.00186554 |

| | | | | | | | |
|---|---|---|---|---|---|---|---|
| Fatigue | LAAS | rs34299746 | T | G | 2.30E-10 | -0.0112145 | 0.00176963 |
| Fatigue | LAAS | rs34404106 | G | A | 4.80E-08 | -0.0104331 | 0.00191174 |
| Fatigue | LAAS | rs3905238 | G | A | 6.60E-09 | -0.0103144 | 0.0017781 |
| Fatigue | LAAS | rs426444 | A | T | 1.20E-08 | -0.010648 | 0.00186685 |
| Fatigue | LAAS | rs4265114 | C | A | 3.10E-08 | 0.00965894 | 0.00174509 |
| Fatigue | LAAS | rs440712 | A | G | 3.30E-08 | 0.010018 | 0.00181335 |
| Fatigue | LAAS | rs4536858 | G | A | 1.80E-09 | -0.0108444 | 0.00180229 |
| Fatigue | LAAS | rs4701803 | T | C | 2.30E-08 | -0.00985094 | 0.00176256 |
| Fatigue | LAAS | rs55761273 | G | A | 4.70E-08 | 0.0135133 | 0.00247427 |
| Fatigue | LAAS | rs55889794 | C | T | 1.70E-08 | 0.0294965 | 0.00523235 |
| Fatigue | LAAS | rs56096694 | T | C | 3.20E-08 | 0.0102745 | 0.00185768 |
| Fatigue | LAAS | rs56203712 | G | A | 3.60E-08 | -0.0116093 | 0.00210732 |
| Fatigue | LAAS | rs56226325 | T | C | 3.50E-08 | -0.0132474 | 0.00240298 |
| Fatigue | LAAS | rs589092 | G | A | 1.00E-08 | -0.0100074 | 0.00174664 |
| Fatigue | LAAS | rs6122735 | T | C | 1.80E-09 | 0.010716 | 0.00178282 |
| Fatigue | LAAS | rs62097947 | T | A | 3.40E-08 | 0.00986865 | 0.00178718 |
| Fatigue | LAAS | rs623025 | C | T | 1.70E-08 | 0.011296 | 0.0020034 |
| Fatigue | LAAS | rs6795061 | C | T | 2.00E-08 | 0.0109787 | 0.00195759 |
| Fatigue | LAAS | rs7035657 | A | T | 4.90E-08 | 0.013833 | 0.00253576 |
| Fatigue | LAAS | rs77087420 | G | A | 5.40E-09 | -0.0223936 | 0.00383847 |
| Fatigue | LAAS | rs948537 | T | C | 2.30E-08 | 0.0117334 | 0.00209935 |
| Fatigue | CEI | rs10041141 | C | T | 1.50E-08 | -0.00993469 | 0.00175377 |
| Fatigue | CEI | rs10158289 | G | A | 3.00E-08 | -0.00972082 | 0.00175356 |
| Fatigue | CEI | rs10438710 | G | A | 4.80E-13 | -0.0157706 | 0.00218063 |
| Fatigue | CEI | rs10502172 | T | C | 4.10E-08 | -0.00956779 | 0.00174386 |
| Fatigue | CEI | rs10741305 | C | T | 3.90E-08 | 0.0096179 | 0.00174982 |

| | | | | | | | |
|---|---|---|---|---|---|---|---|
| Fatigue | CEI | rs10858024 | A | C | 3.40E-08 | 0.00977954 | 0.00177199 |
| Fatigue | CEI | rs10906187 | G | A | 6.00E-10 | 0.0111693 | 0.00180391 |
| Fatigue | CEI | rs11066368 | A | G | 5.20E-09 | 0.0105112 | 0.00179997 |
| Fatigue | CEI | rs113081539 | T | C | 2.50E-09 | 0.0202856 | 0.00340099 |
| Fatigue | CEI | rs11620836 | T | C | 9.20E-10 | -0.0124167 | 0.00202811 |
| Fatigue | CEI | rs11992983 | A | G | 1.70E-08 | 0.00994216 | 0.00176162 |
| Fatigue | CEI | rs12635614 | A | G | 3.90E-08 | 0.00959086 | 0.00174511 |
| Fatigue | CEI | rs139498 | C | G | 1.10E-08 | -0.0100756 | 0.00176391 |
| Fatigue | CEI | rs161645 | G | A | 2.30E-08 | -0.0104122 | 0.00186423 |
| Fatigue | CEI | rs1647394 | T | C | 4.10E-08 | 0.00970963 | 0.00176921 |
| Fatigue | CEI | rs17374635 | C | T | 4.40E-08 | -0.0103017 | 0.00188205 |
| Fatigue | CEI | rs200992 | G | A | 2.20E-08 | -0.0151129 | 0.00270229 |
| Fatigue | CEI | rs2186710 | G | C | 1.40E-08 | 0.0101419 | 0.00178599 |
| Fatigue | CEI | rs2938134 | A | C | 4.90E-08 | -0.010173 | 0.00186554 |
| Fatigue | CEI | rs34299746 | T | G | 2.30E-10 | -0.0112145 | 0.00176963 |
| Fatigue | CEI | rs34404106 | G | A | 4.80E-08 | -0.0104331 | 0.00191174 |
| Fatigue | CEI | rs3905238 | G | A | 6.60E-09 | -0.0103144 | 0.0017781 |
| Fatigue | CEI | rs426444 | A | T | 1.20E-08 | -0.010648 | 0.00186685 |
| Fatigue | CEI | rs4265114 | C | A | 3.10E-08 | 0.00965894 | 0.00174509 |
| Fatigue | CEI | rs440712 | A | G | 3.30E-08 | 0.010018 | 0.00181335 |
| Fatigue | CEI | rs4536858 | G | A | 1.80E-09 | -0.0108444 | 0.00180229 |
| Fatigue | CEI | rs4701803 | T | C | 2.30E-08 | -0.00985094 | 0.00176256 |
| Fatigue | CEI | rs55761273 | G | A | 4.70E-08 | 0.0135133 | 0.00247427 |
| Fatigue | CEI | rs55889794 | C | T | 1.70E-08 | 0.0294965 | 0.00523235 |
| Fatigue | CEI | rs56096694 | T | C | 3.20E-08 | 0.0102745 | 0.00185768 |
| Fatigue | CEI | rs56203712 | G | A | 3.60E-08 | -0.0116093 | 0.00210732 |

| | | | | | | | |
|---|---|---|---|---|---|---|---|
| Fatigue | CEI | rs56226325 | T | C | 3.50E-08 | -0.0132474 | 0.00240298 |
| Fatigue | CEI | rs589092 | G | A | 1.00E-08 | -0.0100074 | 0.00174664 |
| Fatigue | CEI | rs6122735 | T | C | 1.80E-09 | 0.010716 | 0.00178282 |
| Fatigue | CEI | rs62097947 | T | A | 3.40E-08 | 0.00986865 | 0.00178718 |
| Fatigue | CEI | rs623025 | C | T | 1.70E-08 | 0.011296 | 0.0020034 |
| Fatigue | CEI | rs6795061 | C | T | 2.00E-08 | 0.0109787 | 0.00195759 |
| Fatigue | CEI | rs7035657 | A | T | 4.90E-08 | 0.013833 | 0.00253576 |
| Fatigue | CEI | rs77087420 | G | A | 5.40E-09 | -0.0223936 | 0.00383847 |
| Fatigue | CEI | rs948537 | T | C | 2.30E-08 | 0.0117334 | 0.00209935 |
| Fatigue | SVD | rs10041141 | C | T | 1.50E-08 | -0.00993469 | 0.00175377 |
| Fatigue | SVD | rs10158289 | G | A | 3.00E-08 | -0.00972082 | 0.00175356 |
| Fatigue | SVD | rs10438710 | G | A | 4.80E-13 | -0.0157706 | 0.00218063 |
| Fatigue | SVD | rs10502172 | T | C | 4.10E-08 | -0.00956779 | 0.00174386 |
| Fatigue | SVD | rs10741305 | C | T | 3.90E-08 | 0.0096179 | 0.00174982 |
| Fatigue | SVD | rs10858024 | A | C | 3.40E-08 | 0.00977954 | 0.00177199 |
| Fatigue | SVD | rs10906187 | G | A | 6.00E-10 | 0.0111693 | 0.00180391 |
| Fatigue | SVD | rs11066368 | A | G | 5.20E-09 | 0.0105112 | 0.00179997 |
| Fatigue | SVD | rs113081539 | T | C | 2.50E-09 | 0.0202856 | 0.00340099 |
| Fatigue | SVD | rs11620836 | T | C | 9.20E-10 | -0.0124167 | 0.00202811 |
| Fatigue | SVD | rs11992983 | A | G | 1.70E-08 | 0.00994216 | 0.00176162 |
| Fatigue | SVD | rs12635614 | A | G | 3.90E-08 | 0.00959086 | 0.00174511 |
| Fatigue | SVD | rs139498 | C | G | 1.10E-08 | -0.0100756 | 0.00176391 |
| Fatigue | SVD | rs161645 | G | A | 2.30E-08 | -0.0104122 | 0.00186423 |
| Fatigue | SVD | rs1647394 | T | C | 4.10E-08 | 0.00970963 | 0.00176921 |
| Fatigue | SVD | rs17374635 | C | T | 4.40E-08 | -0.0103017 | 0.00188205 |
| Fatigue | SVD | rs200992 | G | A | 2.20E-08 | -0.0151129 | 0.00270229 |

| | | | | | | | |
|---|---|---|---|---|---|---|---|
| Fatigue | SVD | rs2186710 | G | C | 1.40E-08 | 0.0101419 | 0.00178599 |
| Fatigue | SVD | rs2938134 | A | C | 4.90E-08 | -0.010173 | 0.00186554 |
| Fatigue | SVD | rs34299746 | T | G | 2.30E-10 | -0.0112145 | 0.00176963 |
| Fatigue | SVD | rs34404106 | G | A | 4.80E-08 | -0.0104331 | 0.00191174 |
| Fatigue | SVD | rs3905238 | G | A | 6.60E-09 | -0.0103144 | 0.0017781 |
| Fatigue | SVD | rs426444 | A | T | 1.20E-08 | -0.010648 | 0.00186685 |
| Fatigue | SVD | rs4265114 | C | A | 3.10E-08 | 0.00965894 | 0.00174509 |
| Fatigue | SVD | rs440712 | A | G | 3.30E-08 | 0.010018 | 0.00181335 |
| Fatigue | SVD | rs4536858 | G | A | 1.80E-09 | -0.0108444 | 0.00180229 |
| Fatigue | SVD | rs4701803 | T | C | 2.30E-08 | -0.00985094 | 0.00176256 |
| Fatigue | SVD | rs55761273 | G | A | 4.70E-08 | 0.0135133 | 0.00247427 |
| Fatigue | SVD | rs55889794 | C | T | 1.70E-08 | 0.0294965 | 0.00523235 |
| Fatigue | SVD | rs56096694 | T | C | 3.20E-08 | 0.0102745 | 0.00185768 |
| Fatigue | SVD | rs56203712 | G | A | 3.60E-08 | -0.0116093 | 0.00210732 |
| Fatigue | SVD | rs56226325 | T | C | 3.50E-08 | -0.0132474 | 0.00240298 |
| Fatigue | SVD | rs589092 | G | A | 1.00E-08 | -0.0100074 | 0.00174664 |
| Fatigue | SVD | rs6122735 | T | C | 1.80E-09 | 0.010716 | 0.00178282 |
| Fatigue | SVD | rs62097947 | T | A | 3.40E-08 | 0.00986865 | 0.00178718 |
| Fatigue | SVD | rs623025 | C | T | 1.70E-08 | 0.011296 | 0.0020034 |
| Fatigue | SVD | rs6795061 | C | T | 2.00E-08 | 0.0109787 | 0.00195759 |
| Fatigue | SVD | rs7035657 | A | T | 4.90E-08 | 0.013833 | 0.00253576 |
| Fatigue | SVD | rs77087420 | G | A | 5.40E-09 | -0.0223936 | 0.00383847 |
| Fatigue | SVD | rs948537 | T | C | 2.30E-08 | 0.0117334 | 0.00209935 |

**Table S2. Characteristics of selected single nucleotide polymorphisms concerning the causal effect of stroke on malnutrition,and fatigue**

| exposure | outcome | SNP | effect_allele.exposure | other_allele.exposure | pval.exposure | beta.exposure | se.exposure |
|---|---|---|---|---|---|---|---|
| Any stroke | Malnutrition | rs10738606 | T | A | 1.43E-08 | 0.0453 | 0.008 |
| Any stroke | Malnutrition | rs10774624 | A | G | 1.12E-11 | -0.0621 | 0.0091 |
| Any stroke | Malnutrition | rs10883922 | G | C | 1.60E-09 | -0.051 | 0.0084 |
| Any stroke | Malnutrition | rs11984041 | T | C | 6.33E-09 | 0.0786 | 0.0135 |
| Any stroke | Malnutrition | rs12037987 | C | T | 2.73E-08 | 0.071 | 0.0128 |
| Any stroke | Malnutrition | rs12445022 | A | G | 1.05E-10 | 0.0574 | 0.0089 |
| Any stroke | Malnutrition | rs17148926 | C | A | 3.00E-08 | -0.0637 | 0.0115 |
| Any stroke | Malnutrition | rs284278 | G | A | 3.54E-08 | -0.0474 | 0.0086 |
| Any stroke | Malnutrition | rs35443 | C | G | 4.95E-08 | -0.0455 | 0.0083 |
| Any stroke | Malnutrition | rs42031 | T | A | 2.30E-08 | -0.061 | 0.0109 |
| Any stroke | Malnutrition | rs6427304 | G | A | 4.94E-13 | -0.0614 | 0.0085 |
| Any stroke | Malnutrition | rs6901866 | C | T | 3.32E-08 | 0.0463 | 0.0084 |
| Any stroke | Malnutrition | rs6936630 | A | T | 2.37E-08 | 0.0784 | 0.014 |
| Any stroke | Malnutrition | rs7337752 | C | T | 3.27E-08 | 0.0541 | 0.0098 |
| Any stroke | Malnutrition | rs7689945 | T | C | 4.79E-08 | 0.0446 | 0.0082 |
| Any stroke | Malnutrition | rs8103309 | C | T | 3.40E-08 | -0.0501 | 0.0091 |
| Any stroke | Fatigue | rs10738606 | T | A | 1.43E-08 | 0.0453 | 0.008 |
| Any stroke | Fatigue | rs10774624 | A | G | 1.12E-11 | -0.0621 | 0.0091 |
| Any stroke | Fatigue | rs10883922 | G | C | 1.60E-09 | -0.051 | 0.0084 |
| Any stroke | Fatigue | rs11984041 | T | C | 6.33E-09 | 0.0786 | 0.0135 |
| Any stroke | Fatigue | rs12037987 | C | T | 2.73E-08 | 0.071 | 0.0128 |
| Any stroke | Fatigue | rs12445022 | A | G | 1.05E-10 | 0.0574 | 0.0089 |
| Any stroke | Fatigue | rs17148926 | C | A | 3.00E-08 | -0.0637 | 0.0115 |
| Any stroke | Fatigue | rs284278 | G | A | 3.54E-08 | -0.0474 | 0.0086 |

| | | | | | | | |
|---|---|---|---|---|---|---|---|
| Any stroke | Fatigue | rs35443 | C | G | 4.95E-08 | -0.0455 | 0.0083 |
| Any stroke | Fatigue | rs42031 | T | A | 2.30E-08 | -0.061 | 0.0109 |
| Any stroke | Fatigue | rs6427304 | G | A | 4.94E-13 | -0.0614 | 0.0085 |
| Any stroke | Fatigue | rs6901866 | C | T | 3.32E-08 | 0.0463 | 0.0084 |
| Any stroke | Fatigue | rs6936630 | A | T | 2.37E-08 | 0.0784 | 0.014 |
| Any stroke | Fatigue | rs7337752 | C | T | 3.27E-08 | 0.0541 | 0.0098 |
| Any stroke | Fatigue | rs7689945 | T | C | 4.79E-08 | 0.0446 | 0.0082 |
| Any stroke | Fatigue | rs8103309 | C | T | 3.40E-08 | -0.0501 | 0.0091 |
| Any ischemic stroke | Malnutrition | rs1053007 | G | A | 3.58E-08 | 0.0479 | 0.0087 |
| Any ischemic stroke | Malnutrition | rs10849925 | G | A | 2.17E-08 | 0.059 | 0.0105 |
| Any ischemic stroke | Malnutrition | rs11984041 | T | C | 2.60E-08 | 0.0805 | 0.0145 |
| Any ischemic stroke | Malnutrition | rs1448817 | G | A | 2.90E-12 | 0.0638 | 0.0091 |
| Any ischemic stroke | Malnutrition | rs1537371 | A | C | 7.63E-09 | 0.0488 | 0.0084 |
| Any ischemic stroke | Malnutrition | rs17148926 | C | A | 9.96E-09 | -0.0707 | 0.0123 |
| Any ischemic stroke | Malnutrition | rs284278 | G | A | 4.39E-08 | -0.0502 | 0.0092 |
| Any ischemic stroke | Malnutrition | rs28897085 | G | A | 4.90E-08 | 0.0888 | 0.0163 |
| Any ischemic stroke | Malnutrition | rs35436 | T | C | 3.21E-08 | -0.0495 | 0.0089 |
| Any ischemic stroke | Malnutrition | rs42036 | G | C | 2.98E-08 | -0.0596 | 0.0108 |
| Any ischemic stroke | Malnutrition | rs4932370 | A | G | 2.88E-08 | 0.0519 | 0.0094 |
| Any ischemic stroke | Malnutrition | rs6427304 | G | A | 1.17E-09 | -0.0555 | 0.0091 |
| Any ischemic stroke | Malnutrition | rs6825454 | C | T | 7.43E-10 | 0.0564 | 0.0092 |
| Any ischemic stroke | Malnutrition | rs7304841 | C | A | 4.93E-08 | -0.0484 | 0.0089 |
| Any ischemic stroke | Malnutrition | rs74484590 | G | A | 4.63E-08 | 0.0759 | 0.0139 |
| Any ischemic stroke | Malnutrition | rs9316214 | T | C | 2.19E-08 | 0.0575 | 0.0103 |
| Any ischemic stroke | Fatigue | rs1053007 | G | A | 3.58E-08 | 0.0479 | 0.0087 |
| Any ischemic stroke | Fatigue | rs10849925 | G | A | 2.17E-08 | 0.059 | 0.0105 |
| Any ischemic stroke | Fatigue | rs11984041 | T | C | 2.60E-08 | 0.0805 | 0.0145 |
| Any ischemic stroke | Fatigue | rs1448817 | G | A | 2.90E-12 | 0.0638 | 0.0091 |

| | | | | | | | |
|---|---|---|---|---|---|---|---|
| Any ischemic stroke | Fatigue | rs1537371 | A | C | 7.63E-09 | 0.0488 | 0.0084 |
| Any ischemic stroke | Fatigue | rs17148926 | C | A | 9.96E-09 | -0.0707 | 0.0123 |
| Any ischemic stroke | Fatigue | rs284278 | G | A | 4.39E-08 | -0.0502 | 0.0092 |
| Any ischemic stroke | Fatigue | rs28897085 | G | A | 4.90E-08 | 0.0888 | 0.0163 |
| Any ischemic stroke | Fatigue | rs35436 | T | C | 3.21E-08 | -0.0495 | 0.0089 |
| Any ischemic stroke | Fatigue | rs42036 | G | C | 2.98E-08 | -0.0596 | 0.0108 |
| Any ischemic stroke | Fatigue | rs4932370 | A | G | 2.88E-08 | 0.0519 | 0.0094 |
| Any ischemic stroke | Fatigue | rs6427304 | G | A | 1.17E-09 | -0.0555 | 0.0091 |
| Any ischemic stroke | Fatigue | rs6825454 | C | T | 7.43E-10 | 0.0564 | 0.0092 |
| Any ischemic stroke | Fatigue | rs7304841 | C | A | 4.93E-08 | -0.0484 | 0.0089 |
| Any ischemic stroke | Fatigue | rs74484590 | G | A | 4.63E-08 | 0.0759 | 0.0139 |
| Any ischemic stroke | Fatigue | rs9316214 | T | C | 2.19E-08 | 0.0575 | 0.0103 |
| LAAS | Malnutrition | rs10990630 | A | G | 9.35E-08 | -0.1789 | 0.0335 |
| LAAS | Malnutrition | rs12122341 | G | C | 2.03E-07 | 0.1452 | 0.0279 |
| LAAS | Malnutrition | rs12130936 | C | T | 3.82E-07 | 0.4477 | 0.0882 |
| LAAS | Malnutrition | rs137865305 | A | G | 3.47E-07 | 0.5381 | 0.1056 |
| LAAS | Malnutrition | rs470234 | G | T | 2.09E-07 | -0.1868 | 0.036 |
| LAAS | Malnutrition | rs56393506 | T | C | 1.26E-07 | 0.1878 | 0.0355 |
| LAAS | Malnutrition | rs7610618 | T | C | 1.44E-08 | 0.8449 | 0.149 |
| LAAS | Malnutrition | rs7647426 | G | C | 2.29E-07 | 0.3401 | 0.0657 |
| LAAS | Malnutrition | rs77927000 | G | C | 8.92E-10 | 0.2109 | 0.0344 |
| LAAS | Malnutrition | rs78030362 | G | A | 1.53E-07 | 0.2512 | 0.0479 |
| LAAS | Fatigue | rs10990630 | A | G | 9.35E-08 | -0.1789 | 0.0335 |
| LAAS | Fatigue | rs12122341 | G | C | 2.03E-07 | 0.1452 | 0.0279 |
| LAAS | Fatigue | rs12130936 | C | T | 3.82E-07 | 0.4477 | 0.0882 |
| LAAS | Fatigue | rs137865305 | A | G | 3.47E-07 | 0.5381 | 0.1056 |
| LAAS | Fatigue | rs470234 | G | T | 2.09E-07 | -0.1868 | 0.036 |
| LAAS | Fatigue | rs56393506 | T | C | 1.26E-07 | 0.1878 | 0.0355 |

| | | | | | | | |
|---|---|---|---|---|---|---|---|
| LAAS | Fatigue | rs7647426 | G | C | 2.29E-07 | 0.3401 | 0.0657 |
| LAAS | Fatigue | rs77927000 | G | C | 8.92E-10 | 0.2109 | 0.0344 |
| LAAS | Fatigue | rs78030362 | G | A | 1.53E-07 | 0.2512 | 0.0479 |
| CEI | Malnutrition | rs146390073 | T | C | 2.20E-08 | 0.6688 | 0.1195 |
| CEI | Malnutrition | rs17042059 | A | G | 7.79E-34 | 0.3291 | 0.0271 |
| CEI | Malnutrition | rs2595105 | C | T | 2.18E-09 | -0.1269 | 0.0212 |
| CEI | Malnutrition | rs7193343 | C | T | 2.75E-08 | -0.1391 | 0.025 |
| CEI | Malnutrition | rs7680240 | C | A | 1.17E-08 | -0.1163 | 0.0204 |
| CEI | Fatigue | rs146390073 | T | C | 2.20E-08 | 0.6688 | 0.1195 |
| CEI | Fatigue | rs17042059 | A | G | 7.79E-34 | 0.3291 | 0.0271 |
| CEI | Fatigue | rs2595105 | C | T | 2.18E-09 | -0.1269 | 0.0212 |
| CEI | Fatigue | rs7193343 | C | T | 2.75E-08 | -0.1391 | 0.025 |
| CEI | Fatigue | rs7680240 | C | A | 1.17E-08 | -0.1163 | 0.0204 |
| SVD | Malnutrition | rs11196595 | C | T | 4.92E-07 | 0.1658 | 0.033 |
| SVD | Malnutrition | rs12445022 | A | G | 9.26E-08 | 0.1301 | 0.0244 |
| SVD | Malnutrition | rs72932709 | G | A | 4.92E-07 | -0.1812 | 0.036 |
| SVD | Malnutrition | rs76110445 | C | T | 2.45E-07 | 0.1881 | 0.0364 |
| SVD | Malnutrition | rs76576182 | G | A | 1.46E-07 | 0.4436 | 0.0844 |
| SVD | Malnutrition | rs8053128 | A | G | 4.78E-07 | 0.1948 | 0.0387 |
| SVD | Fatigue | rs11196595 | C | T | 4.92E-07 | 0.1658 | 0.033 |
| SVD | Fatigue | rs12445022 | A | G | 9.26E-08 | 0.1301 | 0.0244 |
| SVD | Fatigue | rs72932709 | G | A | 4.92E-07 | -0.1812 | 0.036 |
| SVD | Fatigue | rs76110445 | C | T | 2.45E-07 | 0.1881 | 0.0364 |
| SVD | Fatigue | rs76576182 | G | A | 1.46E-07 | 0.4436 | 0.0844 |
| SVD | Fatigue | rs8053128 | A | G | 4.78E-07 | 0.1948 | 0.0387 |

## Table S3. The single nucleotide polymorphisms corresponds to the gene name

| exposure | outcome | id | gene_ids | gene_names |
|---|---|---|---|---|
| Fatigue | Any stroke | rs17374635 | ENSG00000300155 | - |
| Fatigue | Any stroke | rs10858024 | ENSG00000118655 | DCLRE1B |
| Fatigue | Any stroke | rs10158289 | - | - |
| Fatigue | Any stroke | rs623025 | ENSG00000134369 | NAV1 |
| Fatigue | Any stroke | rs10906187 | ENSG00000183049 | CAMK1D |
| Fatigue | Any stroke | rs1647394 | ENSG00000166793 | YPEL4 |
| Fatigue | Any stroke | rs10741305 | ENSG00000077498 | TYR |
| Fatigue | Any stroke | rs2186710 | ENSG00000247416 | - |
| Fatigue | Any stroke | rs10502172 | ENSG00000149292 | TTC12 |
| Fatigue | Any stroke | rs11066368 | ENSG00000174527 | MYO1H |
| Fatigue | Any stroke | rs11620836 | ENSG00000304903 | - |
| Fatigue | Any stroke | rs440712 | ENSG00000242293 | RPS29P1 |
| Fatigue | Any stroke | rs10438710 | ENSG00000299795 | - |
| Fatigue | Any stroke | rs2938134 | - | - |
| Fatigue | Any stroke | rs948537 | ENSG00000101489 | CELF4 |
| Fatigue | Any stroke | rs62097947 | ENSG00000187323 | DCC |
| Fatigue | Any stroke | rs56096694 | ENSG00000300819 | - |
| Fatigue | Any stroke | rs55761273 | - | - |
| Fatigue | Any stroke | rs6122735 | ENSG00000294533 | - |
| Fatigue | Any stroke | rs139498 | ENSG00000100401 | RANGAP1 |
| Fatigue | Any stroke | rs6795061 | ENSG00000228956 | SATB1-AS1 |
| Fatigue | Any stroke | rs4536858 | ENSG00000270441 | LAMB2P1 |
| Fatigue | Any stroke | rs426444 | ENSG00000287273 | - |
| Fatigue | Any stroke | rs589092 | ENSG00000114054 | PCCB |
| Fatigue | Any stroke | rs12635614 | ENSG00000169760 | NLGN1 |
| Fatigue | Any stroke | rs3905238 | ENSG00000197386 | HTT |
| Fatigue | Any stroke | rs56203712 | ENSG00000168228 | ZCCHC4 |
| Fatigue | Any stroke | rs34404106 | ENSG00000145242 | EPHA5 |
| Fatigue | Any stroke | rs77087420 | ENSG00000138688 | BLTP1 |
| Fatigue | Any stroke | rs4701803 | ENSG00000308020 | - |
| Fatigue | Any stroke | rs161645 | ENSG00000251574 | - |
| Fatigue | Any stroke | rs34299746 | ENSG00000286749 | - |
| Fatigue | Any stroke | rs10041141 | ENSG00000155511 | GRIA1 |
| Fatigue | Any stroke | rs55889794 | ENSG00000291112 | - |
| Fatigue | Any stroke | rs200992 | ENSG00000233822 | H2BC15 |
| Fatigue | Any stroke | rs56226325 | ENSG00000002822 | MAD1L1 |
| Fatigue | Any stroke | rs4265114 | ENSG00000234273 | - |
| Fatigue | Any stroke | rs11992983 | - | - |
| Fatigue | Any stroke | rs7035657 | ENSG00000237385 | - |

Figure S1. Scatter plots, funnel plots, and leave-one-out analysis of the causal relationship between malnutrition and stroke. Any stroke (A-C), any ischemic stroke (D-F), LAAS (G-I), CEI (J-L), and SVD (M-O)

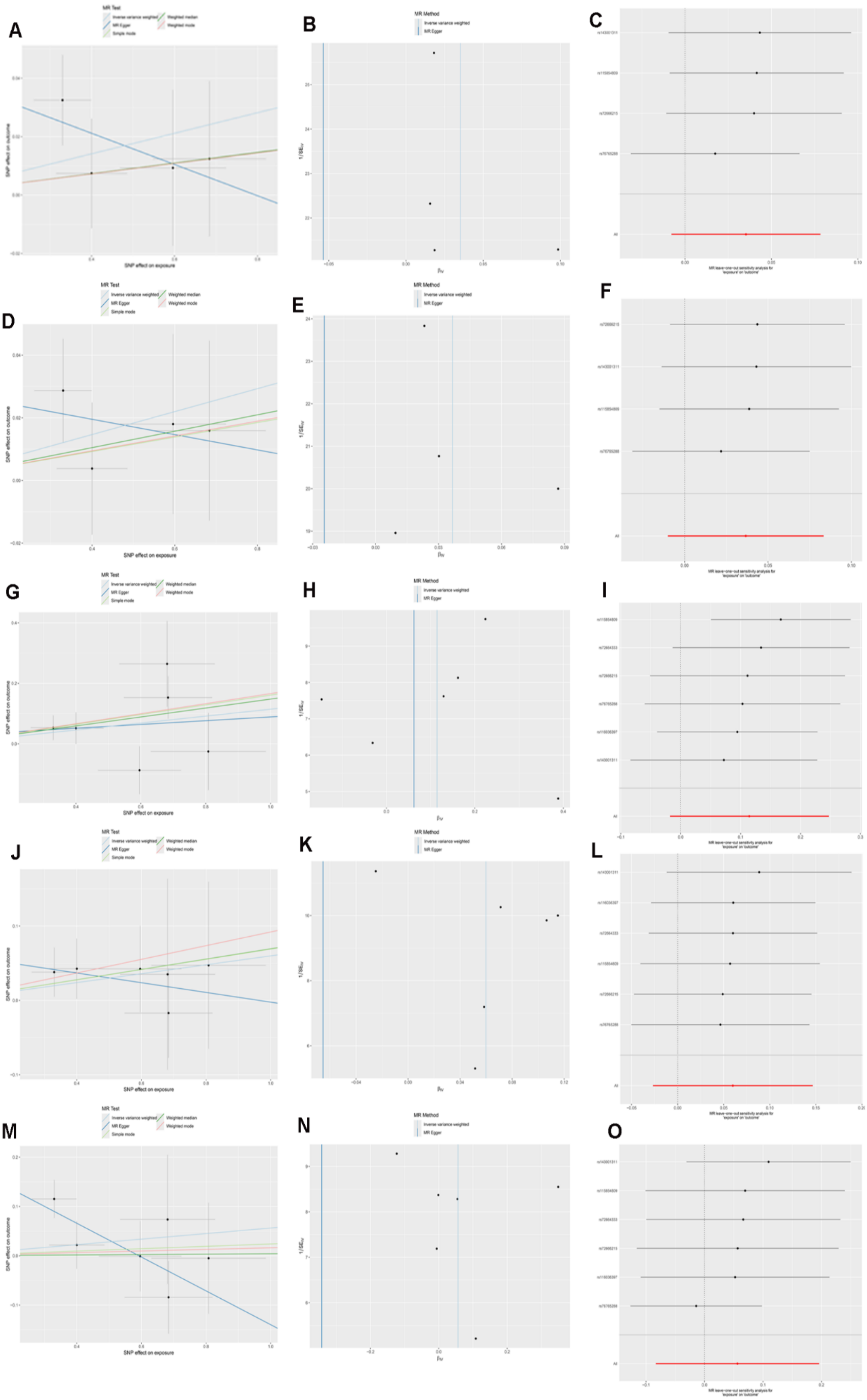

Figure S2. Scatter plots and funnel plots of the causal relationship between frailty and stroke. Any stroke (A-B), any ischemic stroke (C-D), LAAS (E-F), CEI (G-H), and SVD (I-J)

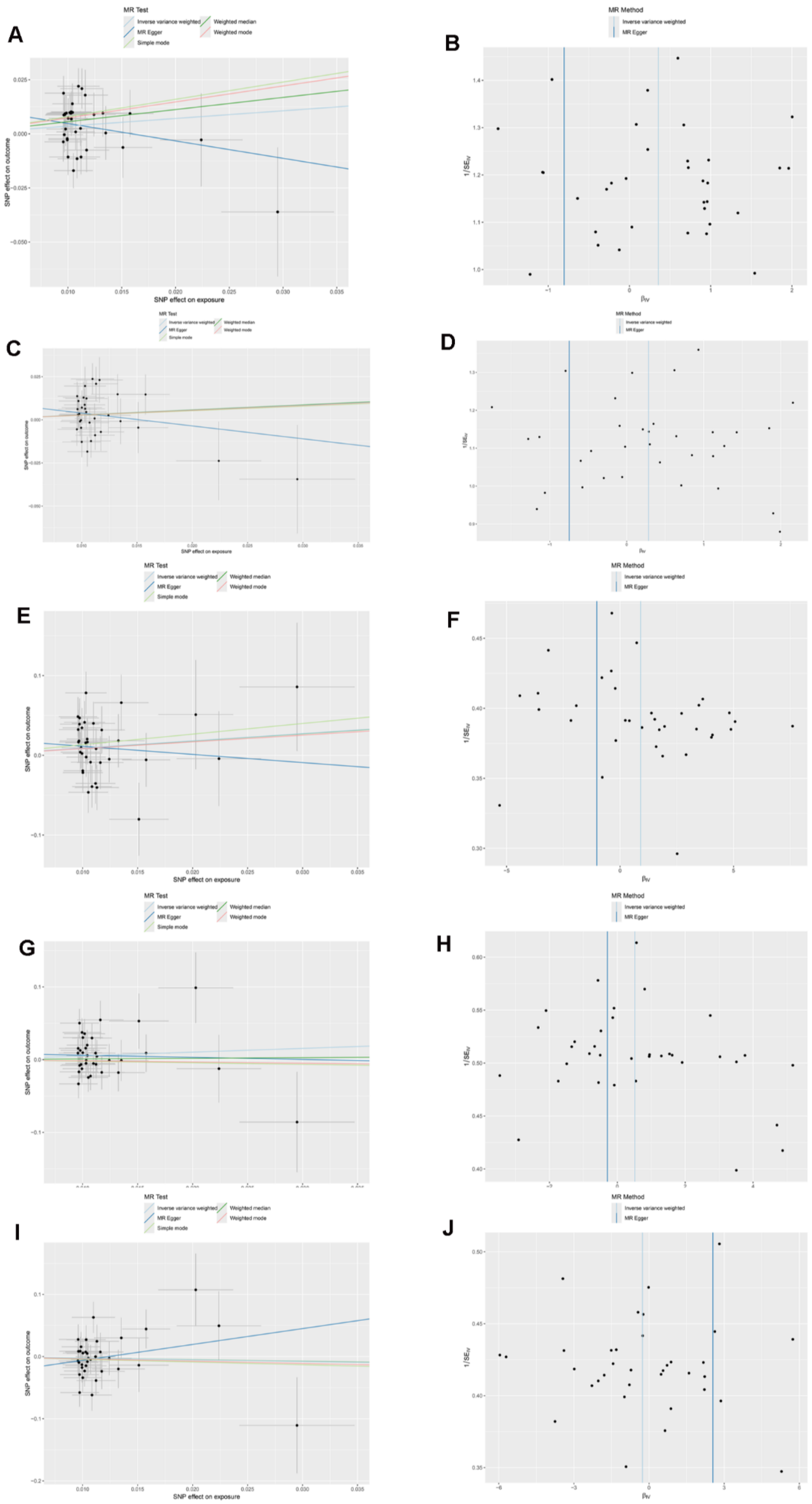

Figure S3. Scatter plots, funnel plots, and leave-one-out analysis of the causal relationship between stroke and malnutrition. Any stroke (A-C), any ischemic stroke (D-F), LAAS (G-I), CEI (J-L), and SVD (M-O)

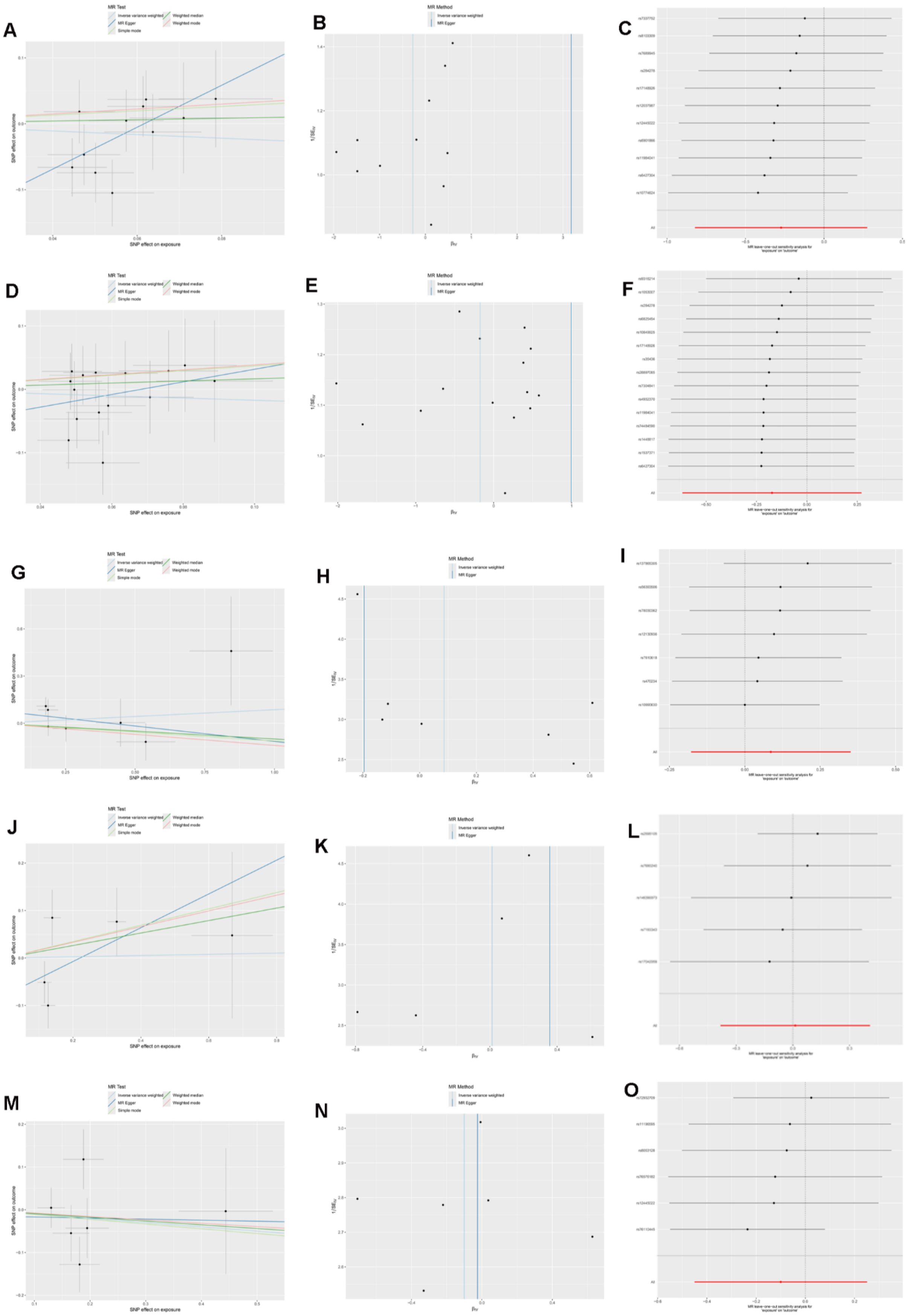

Figure S4. Scatter plots, funnel plots, and leave-one-out analysis of the causal relationship between stroke and frailty. Any stroke (A-C), any ischemic stroke (D-F), LAAS (G-I), CEI (J-L), and SVD (M-O)

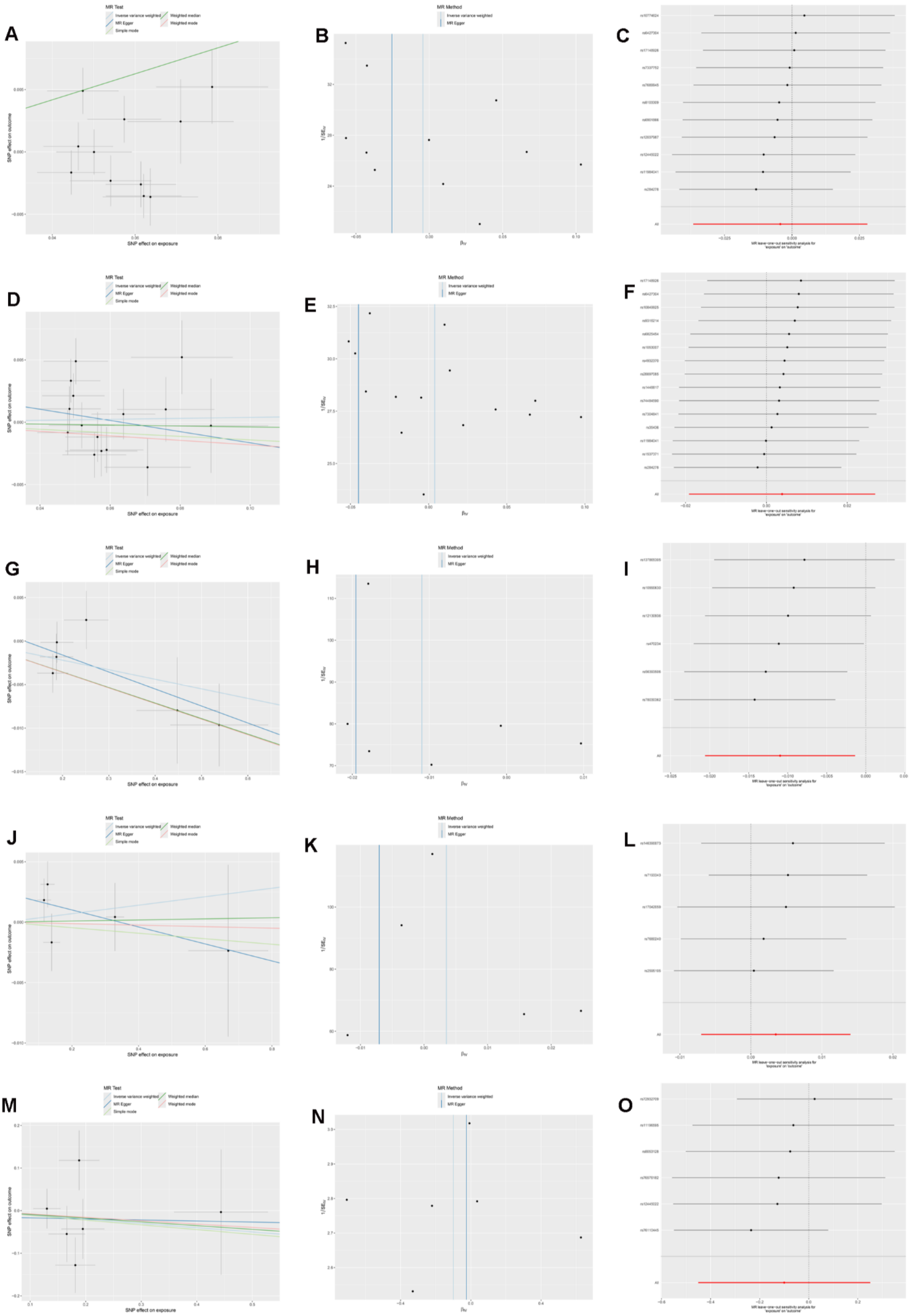

Supplement: Supplementary file 1 [file medi-105-e46894-s001.pdf]
